# Haplotype Estimation from Fuzzy Genotypes Using Penalized Likelihood

**DOI:** 10.1371/journal.pone.0024219

**Published:** 2011-09-08

**Authors:** Hae-Won Uh, Paul H. C. Eilers

**Affiliations:** 1 Department of Medical Statistics and Bioinformatics, Leiden University Medical Center, Leiden, The Netherlands; 2 Erasmus Medical Center, Rotterdam, The Netherlands; 3 Centre for Biosystems Genomics, Wageningen, The Netherlands; Aarhus University, Denmark

## Abstract

The Composite Link Model is a generalization of the generalized linear model in which expected values of observed counts are constructed as a sum of generalized linear components. When combined with penalized likelihood, it provides a powerful and elegant way to estimate haplotype probabilities from observed genotypes. Uncertain (“fuzzy”) genotypes, like those resulting from AFLP scores, can be handled by adding an extra layer to the model. We describe the model and the estimation algorithm. We apply it to a data set of accurate human single nucleotide polymorphism (SNP) and to a data set of fuzzy tomato AFLP scores.

## Introduction

With present-day technology it is hard to economically determine the phase of genotypes, i.e, to allocate SNP alleles to individual chromosomes. This has led to a variety of statistical approaches: certainty is not attainable, but one can estimate the probabilities of the possible haplotypes. It is common to assume that genotypes have been measured accurately. Unfortunately this is not always the case. We will be working with a data set of tomato markers that contains AFLP (amplified fragment length polymorphism) [1]. If we consider a SNP with alleles A and B, the accurate or “crisp” genotypes are AA, AB and BB. AFLP scores frequently contain, “not AA”, “not BB”, or completely missing genotypes; we call these “fuzzy” genotypes. Their existence increases the complexity of the haplotype problem.

In his paper we present a new approach to haplotype probability estimation, or shortly haplotype estimation. It is based on the composite link model (CLM) [2], extended with a penalty. The CLM allows an elegant and powerful formulation, while the penalty stabilizes the computations. In addition one can bring in prior information in an empirical Bayes sense. We apply the model to two data sets: one on human cervical cancer, containing crisp genotypes, the other containing fuzzy AFLP markers, determined in tomatoes.

The problem of haplotype frequency estimation has led to numerous papers and many approaches, but there are two main streams. The first relies on the Expectation-Maximization (EM) algorithm [3] based on a gene counting argument [4]–[6]. At step one, missing phase information is filled in, using current estimates of haplotype frequencies. Then, based on the reconstructed phase, the EM algorithm equates haplotype frequencies to imputed haplotype proportions. This iterative process of imputation and re-estimation is simple and effective. To deal with the increasing number of markers, several approaches have been proposed [7], [8]. The second stream uses the Bayesian approach. Some proposals are based on conjugate priors [9], [10], and another, in the program PHASE, on priors using coalescent models from population genetics [11]. To flexibly capture the clustering of similar haplotypes over short regions, the fastPHASE program uses a hidden Markov model (HMM) to assign phase in each individual and to estimate haplotype frequencies. Bayesian approaches tend to be more accurate than the EM-based methods [12] but incur larger computational costs.

Most methods for haplotype estimation require the genotypes to be accurate or “crisp”, such as AA, AB and BB, that can be achieved by choosing the most probable genotypes. However, forcing the uncertain genotypes to be “crisp” might add another level of uncertainty to the phase ambiguity of haplotypes. Hence, there is a need to develop methods that incorporate uncertain, or “fuzzy” genotypes directly in haplotype estimation. An exception is the GenoSpectrum (GS)-EM algorithm [13].

Maneuvering between the two main streams, we propose an approach based on the penalized composite link model (PCLM) [14]. The composite link model (CLM) of Thompson and Baker [2], is an extension of the generalized linear model (GLM). It turns out that the structure of the CLM catches all elements of our statistical problem in an elegant and powerful way: mating patterns as well as information loss due to the observation of un-phased genotypes. Also the CLM simplifies the notation. The ubiquitous sums of probabilities over compatible sets, that are characteristic for much of the literature in this field, are replaced by concise expressions with matrices and vectors.

Thompson and Baker proposed an algorithm for maximum likelihood fitting of a CLM. We extend it with a penalty on the parameters, with two goals in mind. Firstly the penalty stabilizes the estimation problem, removing the ill condition of estimating equations in larger problems and speeding up convergence [15]. In the second place the penalty achieves that all estimated probabilities will be positive. This is also the case in Bayesian methods, but not in EM algorithms, which will always give zero probability to unobserved (compatible) haplotypes. This may be reasonable in very large samples, but otherwise it is not correct, because it equates unobserved to impossible. The weight of the penalty is optimized by searching for a minimum of Akaike's Information Criterion (AIC) [16]. Additionally we show how to extend the PCLM method to incorporate not only the crisp genotypes (AA, AB, BB) but also fuzzy genotypes: not(AA) or AB
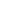
BB, not(BB) or AA
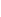
AB, and missing.

In the next section we introduce the model, the penalty and the estimation algorithm. In addition, we discuss natural extensions and additional applications of the model and especially the powerful matrix notation. Application to data from the literature is the subject of the Results section, where we also illustrate our new methods using AFLP marker data. A Discussion concludes the paper.

## Methods

Here we develop the model in three variants. In its most simple form it uses a table of the observed frequencies of all possible crisp genotypes, including zero frequencies. This serves to introduce the composite link model, the penalty, the estimation algorithm and the computation of diagnostics. Then we switch to a variant in which only the observed, crisp, individual genotypes are being used. Finally we show how to generalize to the case of fuzzy genotypes.

### Frequencies of crisp genotypes

Consider 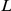 SNPs. The SNP genotype states are coded as the number of copies of the minor (or reference) allele, 0, 1, or 2. A haplotype can be coded as a binary vector of length 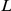, indicating presence or absence of the rare allele. The number of possible haplotypes is 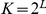. Haplotypes combine in ordered pairs, diplotypes, of which 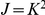 different possibilities exist. In contrast, a genotype is un-phased; it is the sum of the two binary vectors of the haplotypes. Genotypes can be coded as a ternary vector of length 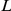, with elements equal to 0, 1 or 2. The number of possible genotypes is 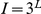. The compatibility between genotypes and diplotypes can be coded by an 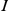 by 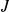 matrix 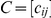. When genotype 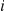 can be formed by the diplotype 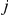, 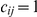; otherwise 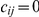. The matrix 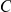 is extremely sparse: 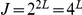 non-zero elements are distributed over 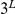 rows and 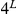 columns. We call 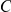 the *composition matrix*.

Let 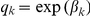 be the probability of haplotype 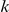. Under random mating, the probability of diplotype 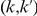 will be 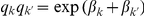. We introduce the 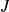 by 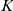 *mating matrix* 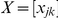. This allows us to write 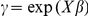, with 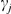 the probability of diplotype 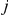. If diplotype 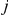 corresponds to the haplotype pair 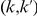, then columns 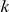 and 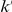 of 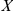 contain a one in row 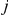. If 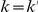, 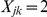. All other elements in that row are zero. Hence 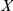 is also very sparse, having at most only two non-zero elements in each row. The construction of the matrices 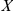 and 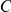 is straightforward.

We show (the transpose of) the matrix 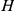 for two SNPs:
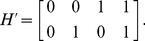
(1)The rows of 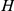 contain the binary notation of the numbers 0 to 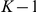. For two SNPs, 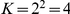 and the transpose of 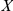 is given by
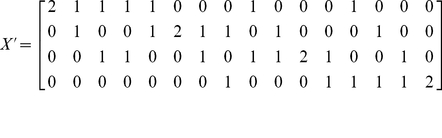
(2)


The 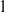
s in the rows of 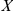 indicate the 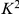 possible ways of forming ordered pairs (the diplotypes) out of 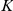 elements (the haplotypes). Finally we show 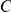 for two SNPs:
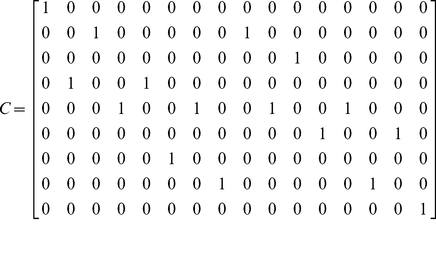
(3)The rows of 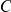 correspond to the genotypes given by 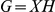:
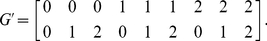
(4)Each row of 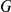 gives the number of rare alleles per SNP for each diplotype. The elements are interpreted as ternary numbers and translated to decimal numbers from 0 to 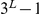. Adding 1 gives the corresponding row of 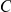 in which a 1 has to be placed, in the column that corresponds to the row of 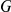.

If we combine mating and composition matrices we have
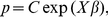
(5)where 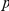 gives the probabilities of the genotypes. This is exactly the composite link model (CLM) of Thompson and Baker [2]. Interestingly, that paper contains a small-scale example, on ABO blood groups, that has the essential flavor of the model we present here. However, to our best knowledge, the CLM has not been adopted by the statistical genetics community.

Estimation of the CLM can be most simply formulated if we assume that 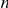 genotypes have been observed and that the absolute frequencies are given as a vector 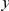 with elements 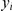, for 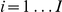. The expected values are given as
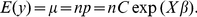
(6)Note that the composition matrix 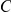 has a row for each possible genotype, whether it was observed or not. Also some elements of the count vector 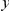 can be zero, reflecting unobserved genotypes. It is not allowed to drop the zero frequency observations: the zeros carry information about the probabilities.

The Poisson log-likelihood is
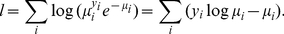
(7)Thompson and Baker show that the GLM scoring algorithm applies, with a modified design matrix, leading to the following iterations:
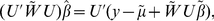
(8)where a tilde, as in 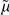 indicates an approximation to the solution, 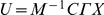, with 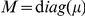, 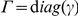 and 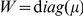. Observe that 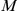 and 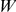 are actually the same, but we prefer this notation to better show the correspondence with the standard GLM fitting.

We extend the CLM with a ridge-type penalty, by forming the penalized log-likelihood
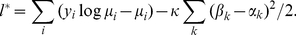
(9)The purpose of the penalty is to push the solution, more or less gently, depending on the value of 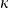, towards a pre-specified distribution 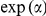. Natural starting values for 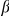 can be based on the assumption of linkage equilibrium for all SNPs. This is also a natural choice for the vector 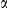 in the penalty: it means that the solution is pushed towards linkage equilibrium. The penalty changes the scoring algorithm only marginally:
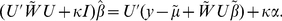
(10)At convergence, standard errors can be obtained for 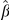 by computing
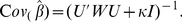
(11)


Two approaches are possible to choose a value for 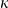, the weight of the penalty. One is to see it simply as a tuning parameter for stability, which should have as low a value as possible, while still giving stability to the estimation process, which means speedier convergence. Alternatively, one can interpret 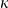 as a model parameter, the inverse of the variance of a prior distribution with mean vector 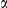. Then it can be optimized using a criterion like AIC. In this context, AIC is defined as 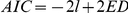, where 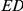 is the effective model dimension, defined as
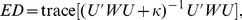
(12)after convergence has been obtained. This choice was inspired by the theory of generalized additive models, as presented by Hastie and Tibshirani [17]. One fits the model for a range of 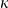
s (say steps of 0.5 or 0.2 on linear grid for 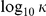) and searches for the minimum of AIC.

To simplify the presentation, we ignored one important practical detail. It is desirable and reasonable to have 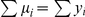. We found that this condition does hold for very high and very low values of 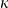 (for our choice of 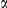, based on linkage equilibrium), but not for values in between. Our solution is to add an (scalar) offset 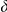, so that the haplotype probabilities are 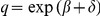. There is no penalty on 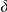.

**Individual genotypes**

In the previous section we modeled expected values of all genotype frequencies (6), including possibly many zeros. As the number of SNPs increases, many genotypes will not be observed and the corresponding elements of 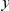 are zero. In fact, unless large sample have been genotyped, most of the elements of 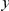 will be zero. We now outline how to more efficiently handle this situation.

Let 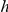 now index an individual, let 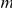 be their number and let 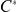 be a new composition matrix in which row 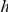 contains the row of 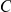 that corresponds to the genotype of individual 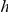. Because different individuals can have the same genotype, some or many of the rows of 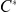 can be identical. With 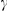 for the probabilities of the diplotypes, 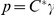 gives the probabilities of the individual genotypes. The log-likelihood is
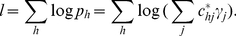
(13)To simplify the presentation, we drop the penalty and consider maximizing this log-likelihood. It is clear that we can make as large as we wish, by making 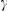 large enough. But because 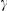 gives the probabilities of all possible diplotypes, we have the condition 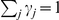. By means of a Lagrange multiplier, 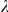, we can incorporate this constraint, so we have to maximize 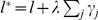.

We skip the details, but it turns out that 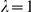. After adding the penalty, we arrive at the same equations as in (10), after replacing 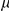 and 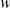 by 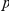. However, to compute the effective dimension we should not use these equations. Instead, after 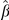 has been found, we compute 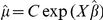, the vector of probabilities of all possible genotypes and insert that into (12).

### Fuzzy genotypes

Genotype information is not always reliable: for some SNPs data may be missing, or only probabilities of the three possible configurations may be available. AFLP markers are an example. A very general model is obtained by introducing an 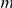 by 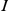 *confusion matrix* 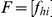 and working with 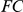 instead of 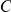. Here the confusion matrix 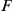 with its elements 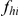 gives the probability that individual observation 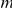 has genotype 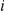. The same device has been used by Kang et al. [13]. The model for the genotype probabilities 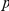 now becomes 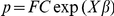. The computations are the same as for the crisp genotypes described earlier, with 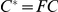.

The matrix 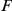 is derived from the fuzzy genotypes of the individuals. Consider one individual and 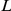 SNPs. Let the rows of the 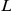 by 3 matrix 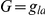 indicate the probabilities of the allelic dose for each SNP for this individual [18]. Assuming independence between the SNPs, the probabilities of all possible genotypes are computed by the repeated Kronecker product of the rows of 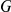. This product determines the row of 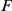 for this individual.

### The power of the CLM notation

The CLM presents a very concise and powerful notation for the haplotype estimation problem. One might say that it works forward from haplotype probabilities to genotype frequencies. There is no need for complicated sums with sets and indices to specify compatibilities between genotypes and haplotype pairs. Various modifications of the model can also be specified concisely. We discuss several examples here.

We introduced a new *confusion matrix* 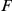, in case that genotype information is not reliable. The use of 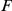 very simply enables us to estimate haplotype frequencies from uncertain genotypes. Completion of the HAPMAP project and the development of imputation software have made large-scale imputation practical. Also software is available for genome-wide association scans with imputed genotypes. To use probabilities of genotypes for haplotype estimation, we expect our algorithm to become a welcome addition to the statistical genetics toolbox.

Generally 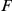 will have more rows than 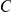 and show the following structure: an identity matrix on top of matrix 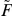, i.e, 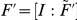. The identity matrix corresponds to all reliably known genotypes (the “crisp” ones). When there are genotypes with one missing SNP, a row in 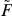 is constructed with all zeroes, except in the three columns corresponding to the three genotypes that are possible to the incomplete genotype at hand. Similarly, two missing SNPs in a genotype would lead to a row in 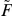 with nine ones. This describes the case when SNPs are either certain or missing. In principle 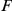 can also be used to code the reliability with which each SNP has been measured by a sequencing instrument.

The model was derived on the assumption of random mating. If one has prior information that this is not the case, the component 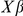 can be modified to 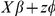. The vector 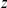 specifies which diplotypes will have their probabilities changed by a factor 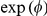, where 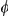 could be specified a priori, or be estimated. More complicated models are possible by changing 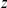 to a matrix 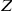 and 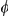 to a vector.

The composite link model can easily be generalized to polypoid organisms such as potatoes, which have four chromosomes. Instead of 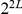 diplotypes we have 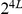 tetratypes, ordered genotypes of alleles. The possible number of genotypes is 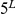 and the 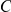 matrix is changed accordingly. This only holds for the special case of full autotetraploidy, the random combination of two chromosomes to form gametes.

## Results

### Human cervical carcinoma

**Figure 1:**
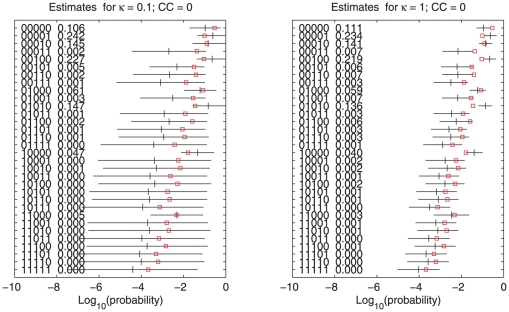
Estimated probabilities and error bars for 32 haplotypes of 5 SNPs in cases (right panel) and controls (left panel) in the cervical carcinoma data. The small squares show the prior probabilities 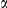. Haplotypes and numerical values of probabilities are shown to the left in each panel.

**Figure 2:**
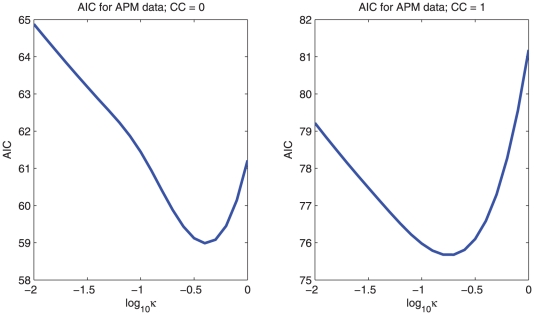
Graph of AIC as a function of 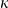
for controls (left) and cases (right).

**Figure 3:**
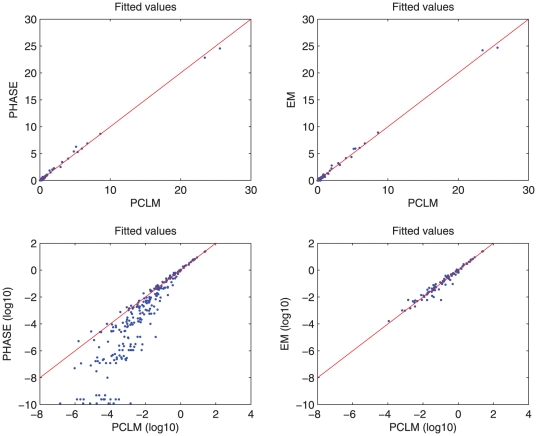
Comparison in haplotype frequency estimation between PCLM and PHASE (left panel), and PCLM and EM (right panel). Top panels: linear scales, bottom panels: logarithmic (base 10) scales. The red lines represent equality.

We first illustrate our methods with data from a case-control study on cervical carcinoma based on “crisp” genotype data. We select 5 SNPs on chromosome 5 and use the control group (122 persons) [19]. Figure 1 shows estimated haplotype probabilities, standard errors and the prior estimates 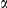. The red squares depict the prior probabilities 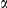, the target values of 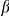, which represent the frequency estimates assuming independency (no linkage disequilibrium (LD)) between the markers. As the shrinkage parameter 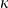 increases, 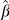 gets nearer to 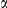. The standard errors decrease when 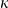 increases. In fact they go to zero for very large 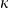; then we have eliminated all uncertainty at the cost of a possibly large bias. This is where AIC comes in: it is an estimate of predictive performance. As Figure 2 shows clear minima are indicated near 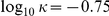 in cases, and 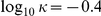 in controls: between the two values used for 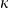 in Figure 1. These figures also indicate that the extent of LD differs between the case and control groups in a candidate region as described in [20]. We analyzed the same data with the PHASE program and SNPHAP (EM algorithm). We found very good correspondence between the two sets of results. This is illustrated in Figure 3. Because PHASE uses Monte Carlo-based computations, the logarithms of smaller probabilities vary appreciably with the length of the Markov Chain and the random starting seed.

### AFLP marker data of tomatoes

**Figure 4:**
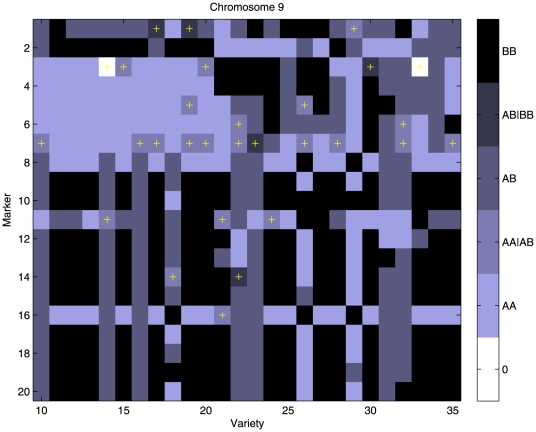
An illustration of tomato AFLP markers. The color bar at the right shows the coding of the fuzzy genotypes. In addition, in the truly fuzzy genotypes a yellow cross has been plotted.

**Figure 5:**
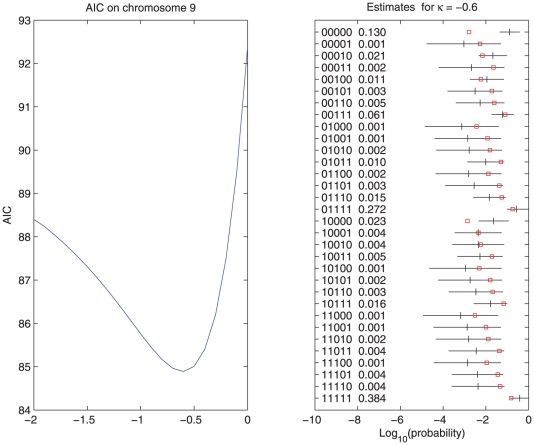
AIC profile and estimated probabilities and error bars for 32 haplotypes of 5 AFLP markers on chromosome 9.

A set of 94 fresh market greenhouse tomato cultivars (mostly hybrids) was provided by a consortium consisting of five international breeding companies. The set of cultivars consisted of total 94 tomato samples. For further details on tomatoes and AFLP markers we refer to van Berloo et al.[1]. Figure 4 illustrates the fuzzy character of the data, for a part of chromosome 9. We selected 5 markers (11 to 15) on chromosome 9. Figure 5 shows estimated haplotype probabilities, standard errors and the prior estimates 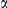 (the red squares).

**Figure 6:**
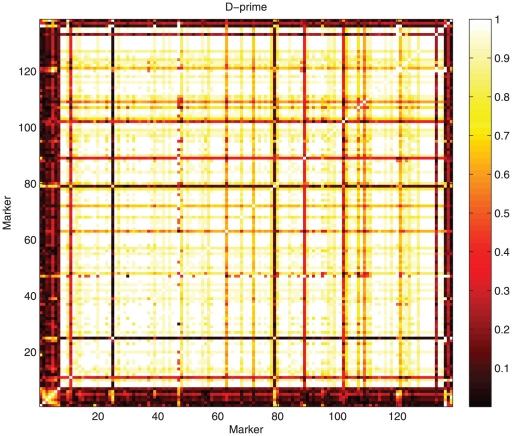
Estimated linkage disequilibrium for all markers on tomato chromosome 9, as measured by 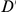.

**Figure 7:**
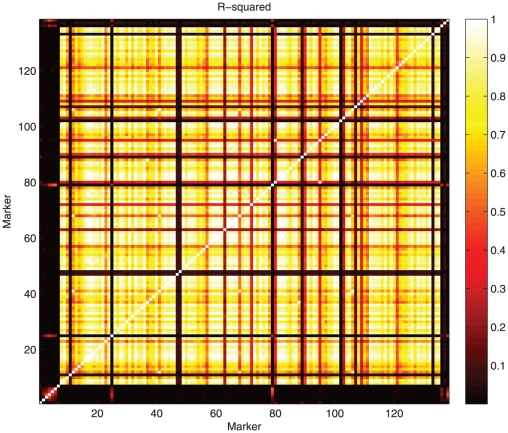
Estimated linkage disequilibrium for all markers on tomato chromosome 9, as measured by 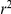.

Testing for Hardy-Weinberg equilibrium (HWE) can be interpreted as haplotype estimation with only one SNP. The penalty can be dropped in this case. In a similar way, investigating linkage disequilibrium (LD) between two SNPs can be approached as haplotype probability estimation too. Again the penalty plays a minor role. We illustrate this with AFLP markers on chromosome 1 and 9 (Figure 6 for 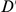 and Figure 7 for 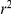. The visual impressions one gets are quite different: 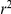 seems to show more detail in the higher regions, while the opposite is true for 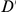. Maybe some kind of rank transform might be desirable, but we consider that discussion as outside the scope of this paper.

**Figure 8:**
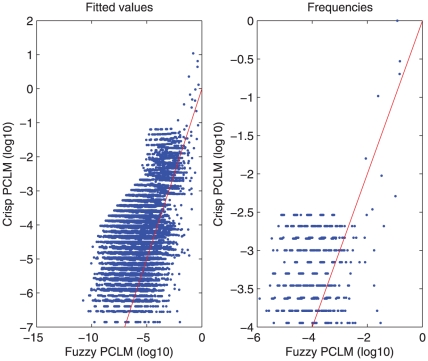
Comparison in haplotype frequency estimation between “fuzzy” PCLM (left panel), and “crisp” PCLM (right panel) in logarithmic (base 10) scales. The red lines represent equality. The “crisp” PCLM shows the results by not correctly handling genotype uncertainty.

When using conventional software, one may code the fuzzy genotypes, AB
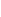
BB and AA
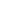
AB, as missing. For comparison of this and our approach, we constructed haplotypes from the 10 SNPs on chromosome 9. With crisp genotype data we found good correspondence between the results obtained by Bayesian, EM and PCLM methods (Figure 3). Figure 8 shows the discrepancies caused by not correctly handling genotype uncertainty.

## Discussion

The penalized composite link model is an elegant and powerful approach to haplotype probability estimation. It provides stable and fast estimation and allows straightforward diagnostic estimation like standard errors.

The penalty has been interpreted mainly as a computational device, to improve stability and to speed up convergence. There is a rapidly growing literature on the relationships between penalties and mixed models. These ideas might be fruitfully transplanted to haplotype estimation. Suppose that a stratifying factor were available. In a traditional approach, we could estimate haplotype probabilities for each stratum separately, or for all strata together. A hierarchical model would postulate a shared distribution, with logarithm 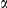 and a penalty for each stratum would allow its 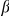 not to deviate too much from 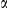. AIC can be used to optimize penalty parameters. In analogy to multi-level GLM, multilevel penalized CLM can be used for hierarchical haplotype probability models.

Unfortunately, our experiments showed that the penalty does not eliminate potential local maxima of the likelihood. Our present tactic is to simply ignore this, to start from the initial solution that reflects linkage equilibrium, and to accept the final estimate. Although it is a “folk theorem” that multiple maxima can occur, it is hard to find documented cases for experimental data. We had to simulate rather extreme data sets to observe multiple maxima.

The algorithm lends itself to the partition-ligation approach [8]. One fits the model to small blocks of SNPs, eliminates the haplotypes with small probabilities, say less than 0.01, and combines adjacent block in pairs, computing the cartesian products of the 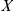 and 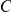 matrices. The products of the block probabilities can be used as starting values for probability vector of the combined block.

The matrices 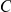 and 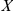 are extremely sparse and in any problem of realistic size they quickly would become too large to fit in computer memory. In our Matlab implementation (available on request) we take advantage of the built-in sparse matrix facilities. In other languages more work might be needed. One possible approach is to store lists of the indices of the non-zero elements and compute indexed sums to get at 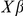, 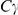 and 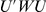 (the latter matrix generally is non-sparse). The system of scoring equations contains 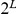 equations, with 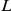 the number of SNPs. A practical limit lies at 10 to 12 SNPs, if these equations are formed and solved explicitly.

In our experience the scoring algorithm is not always stable. Therefore we check whether the proposed update for 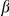 indeed lowers the penalized likelihood. If it does not, we halve the step in the direction 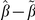. This correction is repeated if needed.

We plan to develop methods to quantify the loss of information due to both genotype uncertainty and phase uncertainty in the context of the PCLM [21].

We already mentioned the extension of the model to haplotype estimation for tetraploid organisms. Examples are plant species such as potato, sugarcane and rose. Due to the flexile framework using the composite link model, our proposed method can straightforwardly be generalized. Our first experiments in this direction have shown favorable results.
